# Editorial: Environmental impacts in domestic birds: towards homeostasis, efficiency and well-being

**DOI:** 10.3389/fphys.2023.1281632

**Published:** 2023-09-01

**Authors:** Shawna Weimer, Gregory S. Fraley, Sara Orlowski, Darrin Karcher, Gregory Archer, Krystyna Pierzchała-Koziec, Colin G. Scanes

**Affiliations:** ^1^ Center for Food Animal Wellbeing, University of Arkansas, Fayetteville, AR, United States; ^2^ Center of Excellence in Poultry Science, University of Arkansas, Fayetteville, AR, United States; ^3^ Department of Animal Sciences, Purdue University, West Lafayette, IN, United States; ^4^ Department of Poultry Science, Texas A&M University, College Station, TX, United States; ^5^ Department of Animal Physiology and Endocrinology, University of Agriculture, Kraków, Poland; ^6^ Biological Science, University of Wisconsin Milwaukee, Milwaukee, WI, United States

**Keywords:** well-being, welfare, stress, homeostasis, chicken

A *sine qua non* is that environmental stressors exert a negative effect on animals via the hypothalamo-pituitary-adrenal axis. Such stresses include cold stress, heat stress, high stocking density, feed restriction environmental and pollutants (Akinyemi and Adewole, 2021). This Research Topic provides a compendium of 11 papers and reviews covering aspects of environmental impacts in domestic birds: towards homeostasis, efficiency and wellbeing. Effects of the following environmental factors were investigated: heat stress, lighting (intensity or duration), isolation stress, pathogens, and pesticides with both physiological and/or behavioral responses determined.

Geoffrey Harris developed the schema of control of the release of anterior pituitary hormones by hypothalamic releasing hormones passing from terminals in the median eminence of the hypothalamus in the hypophyseal portal blood vessels Watts (2015). Arguably implicit to the model were the views that releasing hormones would be specific for individual anterior pituitary hormones and that there would be one, or perhaps two (one stimulatory and one inhibitory), releasing hormones per anterior pituitary hormone. An example of such a control system is the hypothalamo-pituitary- adrenocortical axis with corticotropin releasing hormone (CRH) binding to CRH receptors 1 (CRH-R1) stimulating the release of adrenocorticotropic hormone (ACTH) which, in turn, stimulates the synthesis of glucocorticoids (Kang and Kuenzel, 2014). In addition to this system, there are other neuropeptides participating in the responses to stressors including met-enkephalin and related peptides (Pierzchała-Koziec and Scanes, 2023).

**FIGURE 1:**
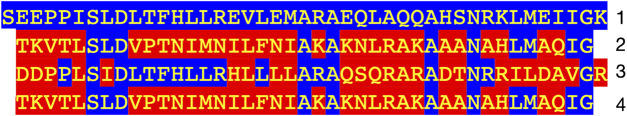
Corticotropin releasing hormone (CRH) family members in chickens: 1—CRH 1 (Vandenborne et al., 2005), 2—CRH 2 (Genbank accession KU887752), 3—urocortin (Genbank accession XM_046939396), 4—urocortin 3 (Grommen et al., 2017). Blue indicates structure of CRH 1 or identical amino acids in other peptides. Red indicates differences to CRH 1.

There are multiple members of the CRH family of genes/peptides across the vertebrates: CRH in eutherian mammals (CRH 1 in non-eutherian mammal and other vertebrates), CRH 2 in multiple vertebrate groups but neither in eutherian mammals nor teleost fish, urocortin (UCN) 1, 2, and 3 (Seasholtz et al., 2002; Cardoso et al., 2016); the last common ancestor between these vertebrate classes existing 290 million years ago (Davis et al., 2012). In chickens, the expressed precursors of four peptides have been identified: CRH 1 (Vandenborne et al., 2005), CRH 2 (Genbank accession KU887752), urocortin (Genbank accession XM_046939396), and urocortin 3 (Grommen et al., 2017) (for sequences of the chicken members of the CRH family of peptides see Figure 1). Across the vertebrates, there are two forms of CRH with CRH 2 having been reported in multiple vertebrates including platypus and opossum (class: Mammalia), lizard (class: Reptilia), coelacanth (class: Actinistia) spotted gar (class: Actinopterygii), elephant shark (class: Chondrichthyes) (Cardoso et al., 2016) together with chicken (class: Aves) (Genbank accession KU887752). In the present Research Topic, there are marked changes in expression of CRH 2 in several hypothalamic nuclei in chickens exposed to the stress of restraint (Kadhim and Kuenzel); “*this being first report in any vertebrate species that following a stressor there occurs significant increases of CRH2 gene expression in two neural structures that play a role in regulating the stress response, the nucleus of the hippocampal commissure and the paraventicular hypothalamic nucleus, resulting in a sustained increase in the stress hormone, corticosterone*” (W.J. Kunzel, personal communication).

There is increasing information of gene expression in neurons within the hypothalamic stress related nuclei in birds. For instance, the premammillary (PMM) nucleus contains neurons expressing enzymes required for the synthesis of dopamine and melatonin together with expression of the light sensitive opsin (Kang). This is consistent with neurons in the premammillary being dual sensory-neurosecretory units (Kang). Moreover, there was greater expression of tryptophan hydroxylase 2, tyrosine hydroxylase and glucocorticoid receptor in the ventral tegmental area in birds raised on low that higher light intensity or on variable light intensity (Kang et al.).

There is cross-talk between the hypothalamo pituitary adrenocortical and thyroid axes in birds with thyrotropin releasing hormone (TRH) receptor 2 present in avian thyrotropes. Chicken thyrotropes not only express CRH-R2 but also both CRH (CRH 1) and urocortin 3 (another ligand for the CRH-R) stimulate thyrotropin release (De Groef et al., 2003). Somatostatin inhibits thyrotropin release in response to either TRH or CRH (De Groef et al., 2005). In the present Research Topic, immobilization stress is demonstrated to induce increases in circulating concentrations of corticosterone and the expression of thyrotropin β subunit in the anterior pituitary gland together with that of the TRH receptor 1 and 3 in the anterior pituitary gland of chickens (Kadhim and Kuenzel).

Pathogens represent a stressor affecting poultry. There were marked differences in ileal and cecal microbiome between conventional broiler chickens and slow growing birds and following *salmonella* challenge (Sheets et al.). *Salmonella* challenge was associated with depressed growth rate in convention broiler chicks but not slow growing chickens (Snyder et al.).

Heat stress adversely affects poultry. In this Research Topic, differences in the responses to heat stress were reported between unselected broiler type chickens and chickens highly selected for growth (Brugaletta et al.). Both growth and feed intake were decreased in 1995 random bred (equivalent to broiler chickens of the 1990s), and fast growing modern random bred (a mixture of the broilers of the 2020s) subjected to heat stress but did not influence growth or feed intake in either Jungle fowl, Athens Canadian Random Bred (equivalent to broiler chickens of the 1950s) (Brugaletta et al.). However, hypothalamic expression of feeding related neither neuropeptides nor their receptors were not affected by elevated environmental temperatures (Brugaletta et al.).

Heat stress was accompanied by increases in the plasma concentration of both corticosterone and cortisol but decreases in egg production in domestic female ducks (Oluwagbenga et al.). Body condition scoring rubric for welfare assessment was employed also to assess the welfare of the birds (Oluwagbenga et al.). Heat stress depressed welfare as assessed by feather quality and cleaniless together with foot pad condition (Oluwagbenga et al.). Chronic heat stress was accompanied by increases in the norepinephrine concentrations in the egg albumin but not yolk of domestic ducks However, there were no effect of chronic heat stress on the concentrations in the norepinephrine precursor, L-3,4-dihydroxyphenylalanine (l-dopa) in either the albumin and yolk (Lyte et al.).

Domesticated birds respond to two characteristics of light, namely, duration and intensity. Transfer of mountain ducks from a long-daylength (17L5D) to continuous lighting (24L:0D) was accompanied by increased plasma concentrations of gonadotropin releasing hormone (GnRH), luteinizing hormone (LH), prolactin, progesterone (P_4_), testosterone and estradiol (E_2_) (Liufu et al.). Hypothalamic expression of TRH was increased in ducks on 24L:0D but decreased in 8L:16D (Liufu et al.). Similarly, expression of TSH β sub-unit in the anterior pituitary gland was increased in ducks on 24L:0D but decreased in 8L:16D (Liufu et al.). It is questioned whether continuous lighting is stressful.

Light intensity influences birds. For instance, the incidence of dust bathing was greater in broiler chickens raised with a variable light intensity in different parts of the pen compared to either those raised on either 5 or 20 lux light intensity (Kang et al.). Moreover, the responses to a novel object test were greater in young broiler chickens raised under a low light intensity compared to a higher light intensity which in turn were greater than birds under a variable lighting regimen (Kang et al.). There was greater expression of tryptophan hydroxylase 2 in both the caudal raphe nucleus and the dorsal raphe nucleus (Kang et al.). There were also differences with birds on natural lighting (Kang et al.). It is also questioned whether low and/or high light intensities and/or an environment with little differences in light intensity are stressful.

Fear is a stressor. Line differences in fear related behaviors in laying hens such as latency following tonic immobility and vocalization following isolation stress (Brown et al.). In addition, the were line/genetic differences in plasma concentrations of corticosterone, heterophil to lymphocyte ratio and physical asymmetry (Brown et al.).

Chickens are used as models for the effects of potential toxicants in the environment on reproduction. An example of this is a definitive study by Fréville et al. Glyphosate is a widely used herbicide and is consequently present in the environment. There were multiple effects of the addition of glyphosate-based herbicide to the feed of laying hens including increases in the relative weight of the gizzard, in the plasma concentrations of the oxidative stress parameter, thiobarbituric acid reactive substances (TBARS) in the liver and muscle, plasma concentrations of aminomethylphosphonic acid (AMPA) and cecal concentrations of short chain fatty acids (Fréville et al.). Moreover, in a companion paper, the same group reports differences in growth rate, circulating concentrations of triglyceride and the adipokinin, chemerin, together with abdominal adiposity and behaviors in the progeny of hens that had previously been treated with glyphosate-based herbicide compared to control hens (Estienne et al., 2023).
